# Evolutionary Developmental Biology and Human Language Evolution: Constraints on Adaptation

**DOI:** 10.1007/s11692-012-9162-y

**Published:** 2012-03-07

**Authors:** W. Tecumseh Fitch

**Affiliations:** Department of Cognitive Biology, Faculty of Life Sciences, University of Vienna, Vienna, Austria

**Keywords:** Evo-devo, Language evolution, Adaptation, Exaptation, Constraints, Spandrel, Phenotypic plasticity

## Abstract

A tension has long existed between those biologists who emphasize the importance of adaptation by natural selection and those who highlight the role of phylogenetic and developmental constraints on organismal form and function. This contrast has been particularly noticeable in recent debates concerning the evolution of human language. Darwin himself acknowledged the existence and importance of both of these, and a long line of biologists have followed him in seeing, in the concept of “descent with modification”, a framework naturally able to incorporate both adaptation and constraint. Today, the integrated perspective of modern evolutionary developmental biology (“evo-devo”) allows a more subtle and pluralistic approach to these traditional questions, and has provided several examples where the traditional notion of “constraint” can be cashed out in specific, mechanistic terms. This integrated viewpoint is particularly relevant to the evolution of the multiple mechanisms underlying human language, because of the short time available for novel aspects of these mechanisms to evolve and be optimized. Comparative data indicate that many cognitive aspects of human language predate humans, suggesting that pre-adaptation and exaptation have played important roles in language evolution. Thus, substantial components of what many linguists call “Universal Grammar” predate language itself. However, at least some of these older mechanisms have been combined in ways that generate true novelty. I suggest that we can insightfully exploit major steps forward in our understanding of evolution and development, to gain a richer understanding of the principles that underlie human language evolution.

## Introduction

*Although our brain represents the main adaptive feature of our species, what it is adapted to is not clear at all.*

François Jacob (1977)

After many years of neglect, the evolution of human language has recently become a very active field of interdisciplinary research. Biologists, linguists, psychologists, neuroscientists, anthropologists, computer modelers, and many others have begun working together, and considerable empirical progress has been made on some components of language (for example, speech perception and production, or the neural basis of syntax perception). A general conception that language must be conceived as composed of multiple semi-independent components, rather than a monolithic “organ”, has begun to take hold. Along with this, various conceptions of “protolanguage” have been clarified: hypothetical systems, during past periods of hominin evolution, that possessed some but not all of the mechanisms typifying modern language. Today, various long standing approaches to language evolution can be conceptualized as different models of protolanguage, and arranged in sequence to offer a specific trajectory from our extinct common ancestor with chimpanzees to modern *Homo sapiens* (cf. Fitch 2010). In particular, it is increasingly widely agreed that *some* components of language have homologues or analogues in other species, and can thus be studied comparatively, while others are probably unique to our species. The increasing focus today is on testing the various hypotheses on the market with diverse empirical data, rather than the speculative story-telling for which the field was so long ridiculed.

Despite some real progress, the field is nonetheless characterized by major unresolved controversies, and one suite of issues in particular will occupy me here. Is language an adaptation, marked as such by its many superbly functional details, which develop optimally in our species and in no other (Pinker and Bloom 1990)? Or is language, in contrast, a hodge-podge of ancient mechanisms, tinkered together into a barely-functional Rube Goldberg device (Jacob 1977), an example of what Francis Crick called a “frozen accident” (Crick 1968)? Is human language best characterized by its continuity with other aspects of cognition and/or communication, and preexisting primate precursors (Hockett and Ascher 1964), or is discontinuity the rule (Premack 2007)? Are some aspects of language, such as the Merge operation of syntax, characterized by near-perfect optimality in a way that cannot be explained via evolutionary “tinkering” (Chomsky 2010)? Is human language basically a special case of a more general, human specific adaptation for shared intentionality and cultural learning (Tomasello et al. 2005)? Such issues have been at the forefront of recent debates about the nature of language and its evolution (Andrews et al. 2002; Hauser et al. 2002; Fitch et al. 2005; Pinker and Jackendoff 2005; Számadó and Szathmary 2006; Botha 2011).

I have suggested elsewhere that each of these viewpoints is likely to be correct for certain components of language, but not for others (Fitch 2010). The appearance of conflict among them is deceiving, for in each case proponents of a specific perspective on language focus on a different component of language. These differences of focus are often obscured by the use of one term, “language”, to refer to different mechanisms or processes. I define “language” as a system which allows virtually *any* thought an organism can conceive to be expressed as a complex signal, and allows others possessing the system to interpret that signal, recreating the original concept. Further, I advocate a *multi-component approach* which sees this capacity as composed of multiple interacting subsystems, rather than a single monolithic whole. This contrasts with the common tendency to seek a single key feature that defines language.

Many components of language will be shared with various other species, but some may be unique derived features of humans (Hauser et al. 2002; Fitch 2005, 2009b). According to current information, several components of human language required substantial evolutionary change during the transition from our last common ancestor with chimpanzees, who lived about 6 million years ago in Africa, to humans. These include adaptations for *signaling* (e.g. vocal imitation), for *semantics* (e.g. advanced Theory of Mind, and a drive to share meanings), and for *syntax* (e.g. recursive combinatorial operations). Scientists court unnecessary confusion and fruitless debate if they fail to distinguish among these different mechanisms, or single out one as the key defining ingredient of language.

In this paper, perhaps controversially, I will suggest that any component of language, even the most novel and apparently adaptive, needs to be characterized within a context of *historical constraints*, deriving from developmental and phylogenetic constraints on form and physiology. Since Darwin, the importance of such constraints has been widely recognized by biologists, and to morphologists and developmental biologists in particular. Thus, to scientists trained in evolutionary biology, much of what I say below may seem obvious. It is nonetheless important to make these points explicitly, when discussing the evolution of cognition, because scientists studying cognition come from diverse disciplinary backgrounds (e.g. psychology, linguistics, neuroscience, anthropology…) and thus may be unaware of the value of a broad and pluralistic approach to evolutionary explanation in which constraints play a central role. This danger is exacerbated by the widespread focus of contemporary evolutionary psychology on adaptive optimization to hypothetical “problems faced by our Pleistocene ancestors” (Tooby and Cosmides 1990; Symons 1992; Pinker 1997; Buss et al. 1998) and a relative reluctance to incorporate historical and developmental constraints into evolutionary theorizing.

In keeping with the evo-devo theme of this issue, I will suggest that now is a particularly opportune time to begin more thoroughly incorporating historical constraints into our thinking about the evolution of cognition, because of the revolution in molecular developmental biology, and the fusion of evolutionary theory and developmental biology that has resulted. Today, for the first time in history, we can begin to ground such relatively vague traditional notions as “phylogenetic constraint” in terms of specific developmental processes that employ well-understood gene networks and molecular interactions. As a massive and unexpected bonus, these developmental genetic processes turn out to be very widely shared among animals, allowing us to gain insights from nematode worms or fruit flies that are directly relevant to human development and evolution (Gehring and Ikeo 1999; Carroll et al. 2005; De Robertis 2008). Biologists are finally in a position to cash out Darwin’s ideas about “correlations of growth”, Bateson’s notions of developmental discontinuity, or Gould’s ideas about allometry, neoteny and exaptation in specific mechanistic terms (cf. Gould 2002). Evo-devo has given substance to these ideas that, although venerable, have long had an uncomfortable whiff of wooliness or even mysticism about them. My goal here is to show that, when trying to make sense of the complex palimpsest that is the modern human brain, cognitive scientists interested in evolution have much to gain by incorporating these insights, and much to lose by ignoring them.

### Constraints and Exaptation in Human Cognitive Evolution

In two seminal papers, Steven Jay Gould and his colleagues clarified several important concepts underpinning what Gould termed a “pluralistic” perspective in evolutionary biology (Gould and Lewontin 1979; Gould and Vrba 1982). These three interlocking concepts are exaptation, constraints and spandrels. While the rhetorical charge of these papers led, initially, to considerable controversy, by now these concepts and this perspective have been integrated into normal “textbook” evolutionary biology (e.g., Ridley 1997). Furthermore, the pluralistic perspective has been embraced profitably by working evolutionary biologists in many fields, including human evolution, generating new testable hypotheses [reviewed in (Andrews et al. 2002; Pievani and Serrelli 2011).

*Exaptation* captures the notion that evolved traits can change their function, being (in Darwin’s terms) co-opted from an old function to some new one. *Constraints* is a covering term for diverse factors that prevent natural selection from fully optimizing a given trait to its function, and that thus restrict, limit, or scaffold the course of evolution and the nature of evolved trait. Because of constraints, selection on one trait may lead to changes in other traits that are not adaptive, but are merely correlated with the selected traits. When such non-adaptive traits appear due to physical or developmental constraints, Gould & Lewontin suggested the term *spandrels*, by analogy to geometrically necessary aspects of architecture. Spandrels in the biological sense are non-adaptive by-products of developmental processes, sometimes present by physical necessity. Exaptation can occur in two forms. In the first, an adaptive structure constructed by natural selection for one purpose can be put to new use—a form of “adaptation recycling”. In contrast, type II exaptations co-opt previously useless spandrels for some use, giving rise to a true novelty. In both cases, it is likely that eventually natural selection further hones and refines certain aspects of the “new” trait, which then constitute *bona fide* adaptations to the new function. Thus there is no *a priori* incompatibility between these concepts: in the pluralistic perspective all of them will play a part in evolutionary explanation, with their respective roles to be sorted out empirically, on a case by case basis.

The broad acceptance of a pluralistic perspective on evolutionary explanation by biologists stands in sharp contrast to recent debates about human cognitive evolution, and language evolution in particular. Receptions run the gamut from the enthusiastic embrace of exaptationist thinking by some (Gould 1991; Tattersall 2004; Chomsky 2010) to skepticism or vigorous rejection by others (Dennett 1995; Buss et al. 1998; Pinker and Jackendoff 2005; Bickerton 2010; Botha 2011). Recent attempts at synthesis have highlighted the need for precise hypotheses and evidentiary standards in evaluating this debate (Andrews et al. 2002; Botha 2011; Fitch 2011a; Pievani and Serrelli 2011).

There can be little doubt from this literature that the value of the pluralistic perspective in understanding human cognition, and language evolution, remains disputed. Part of this is a matter of time: acceptance of this perspective in biology required the specification and empirical testing of clear exaptive hypotheses (e.g., Poe et al*.* 2007). By comparison, the study of human cognitive evolution remains in its infancy. In a step towards this goal, in a companion paper to this one I have specified three specific, testable hypotheses concerning the evolution of spoken language and its neural underpinnings (Fitch 2011a). However, part of the problem seems to be a deeper symptom of interdisciplinary exchange and mutual misunderstanding, particularly of the importance of evolutionary constraints in biological explanation. It is this problem that I focus on here.

### Evo-Devo and the Panglossian Paradigm

A core issue in the ongoing debate about constraints and adaptation in cognitive evolution concerns the proper role of the concept of adaptation by natural selection in evolutionary explanations. One camp, prominent among evolutionary biologists, stresses that adaptation is an “onerous concept” to be invoked only after other explanations (e.g. historical accident, random drift, and the like) have been empirically rejected (Williams 1966b; Gould and Lewontin 1979; Ahouse 1998). The other camp, prominent among evolutionary psychologists, advocates adaptation as a *default assumption* (Dennett 1983; Pinker and Bloom 1990; Dennett 1995; Buss et al. 1998; Andrews et al. 2002; Pinker and Jackendoff 2005). This camp suggests that adaptive hypotheses must be rejected empirically before other non-adaptive alternatives can be entertained. This is an epistemological issue, concerning the proper way to practice evolutionary biology and apply it to the human mind.

Philosopher Daniel Dennett provides a compelling metaphor, based on chess, to support his contention that adaptation in general, and optimal design more specifically, deserve the status of default assumption in evolutionary theory (Dennett 1995). In chess, stronger players often give an advantage to beginners by forfeiting a piece (a knight, bishop or even queen) to help even the game. Imagine, suggests Dennett, that instead the stronger player decides to restrict their possible moves (e.g. no diagonal moves by queens, never moving a piece twice in a row), and write this constraint down at the beginning of play, but does not tell their opponent. How then should the opponent determine what this self-imposed constraint is? By comparing the actual moves to the *optimal* moves, and noting any discrepancies between them. This, argues Dennett, is how evolutionary biologists should proceed as well. Mother Nature does not write down the constraints, and since we cannot “read” the restrictions directly, we should assume that evolution is optimal, until obtaining clear evidence to the contrary. By this argument, we can only obtain evidence for non-adaptive hypotheses by considering and testing all plausible adaptive hypotheses (for further argument and endorsement of this view see Andrews et al. 2002).

There are two core problems with Dennett’s chess metaphor. First, in chess we can assume that the opponents goal is to win the game: we know what “optimality” means. If the player was acting to prolong the game, or with a perverse attachment to rooks, the proposed optimality strategy would fail. In nature, of course, the goal of the trait (its putative adaptive function) is precisely what is being debated. The second problem is the assumption in the metaphor that the opponent cannot read the rules restricting the constrained player. But the core benefit of studying development, particularly in the broad comparative framework favored by modern evo-devo, is that we *can* “read the rules” and understand the constraints by directly studying developmental processes, and also understand historical processes based on phylogenetic inference. Hijacking Dennett’s metaphor, in modern developmental biology we often can peek directly at the constraining rules. When this is possible it is a far more productive way to proceed than laboriously employing the “optimality gambit”.

Thus, I conclude that recent progress in biology supports the traditional viewpoint of (Williams 1966b) that adaptation is an onerous concept to be invoked only after a pluralistic set of plausible non-adaptive hypotheses (chance, constraints, spandrels, exaptation, phenotypic plasticity) have failed. Obviously, a solid evidential basis is necessary to accept or reject *any* scientific hypothesis, and hypotheses about constraints are no exception. But I will argue that we will make far more rapid progress in understanding human cognition by grounding evolutionary hypotheses in developmental, phylogenetic and ontogenetic data than we would by restricting ourselves to adaptationist arguments about optimal function.

### Outline

The rest of this paper has three parts. In the first part, I take a historical perspective to clarify the distinction between adaptation and evolution, and illustrate the fundamental importance of constraints in understanding evolution. Much of the current debate in evolutionary psychology fails to distinguish evolutionary explanations (which include phylogeny, constraint, and developmental bias as key components) from adaptive explanations (which focus nearly exclusively on the optimizing function of natural selection). I argue that well-established biological fields, including molecular biology and functional morphology, provide better role models for future cognitive biology (following, for example, Jacob 1977; Maynard Smith 1978b; Lauder 1990) than does the current incarnation of evolutionary psychology. I illustrate this contention with examples from morphology and genetics, and show that by using a comparative phylogenetic approach, we can distinguish local adaptation to problems specific to a species, from traits reflecting global constraints applicable to a much broader set of organisms. By distinguishing local adaptations, involving immediate problems a species faces in its current environment, from global design constraints (e.g. the *Bauplan* of its phylum), we can make the concepts of adaptation and constraint both more precise, and more explanatory.

In the second half of the paper, I suggest that the clarification of the dual concepts of adaptation and constraint, coupled with a vastly improved understanding of developmental and phylogenetic constraints provided by evo-devo, has important implication for the study of language evolution. This perspective finds its roots in the “tinkering” metaphor of François Jacob (1977), the pluralistic perspective on biological inquiry of Tinbergen (1963), and the acknowledgement of constraints championed by Gould and Lewontin (1979). An evo-devo framework has room for both continuity and change, and can naturally incorporate both key innovations, adaptations sharpened by natural selection, and the phenotypic limitations inherent in our phylogenetic starting point. I initially explore several examples of increasing relevance to human language evolution, considering two aspects of human morphology that provide illustrative examples: bipedalism and the speech apparatus. I then turn to the evolution of human cognition, again providing several well-researched examples from neuroscience. These examples show how cognitive biology can embrace a pluralistic perspective on adaptation, avoid the overly simplistic dichotomies implicit in much current debate in language evolution, and enrich our understanding of language and cognition. I end by sketching an approach to language evolution that incorporates the insights of evo-devo and the mechanistic nature of constraints on evolution, arguing for what I term an “exaptationist” perspective, which sees evolution as a cascade of exaptations and adaptations.

## Part 1: Evolutionary Constraints and Adaptation

### Evolution and Adaptation: A Historical Prelude

*Thus throughout nature almost every part of each living being has probably served, in a slightly modified condition, for diverse purposes, and has acted in the living machinery of many ancient and distinct specific forms* (p 284 Darwin 1877)

Two themes—of common descent and adaptive design—play a recurring role in Charles Darwin’s writings. Darwin provided abundant data supporting the fact of evolution, highlighting the linked themes of “descent with modification” and phylogenetic constraints throughout his writing. He emphasized the many useless “vestiges” or “rudiments” found throughout nature, such as the human vermiform appendix, as clear evidence of constraints on the process of evolution, and evidence against any all-knowing, globally optimizing Creator. As a result of Darwin’s emphasis of these facts, the idea that biological traits, and species, evolve by descent with modification, became widely accepted within 10 years of the publication of the *Origin of Species*.

Darwin’s second core concept was adaptation: that the process of natural selection leads organisms to be remarkably well-suited to their ways of life, and thus “well-designed” in an engineering sense. Unlike constraints and common descent, *this* concept was already widely accepted in Victorian England long before Darwin wrote the *Origin*, because it had been emphasized by the natural theologians as evidence for the existence of a Creator (e.g., Paley 1826). Darwin thus had no need to convince readers of the *existence* of many “remarkable contrivances” which reflect excellent design. In the *Origin*, his purpose instead was to provide a non-deistic *explanation* of such design, which Darwin and Wallace famously provided with their theory of natural selection. Unlike descent with modification, the importance of natural selection was not well-accepted in Darwin’s time, and by the early 1900s natural selection was seen by many scientists as an outdated Victorian idea, with little explanatory power (cf. Ridley 1997). It was not until the neo-Darwinian synthesis of genetics and evolution occurred that natural selection was rescued from disrepute, and elevated to its current status as a prime mover in evolutionary change.

But even this elevation was only partial, and remains a topic of ongoing debate among biologists. For example, today, the widely-accepted “neutral theory” of molecular evolution holds that most variation in protein and DNA sequences is a result of random drift, rather than natural selection (Kimura 1983). However, the degree to which the neutral theory is actually true, rather than a simple null hypothesis to be falsified in many cases, remains debated (cf. Graur and Li 2000; Wagner 2008). More generally, many biologists have emphasized the importance of developmental constraints on the evolutionary process (Gould 1977; Gould and Lewontin 1979; Maynard Smith et al. 1985; Gottlieb 1992; Goodwin 1996) and argued for a need to incorporate developmental biology into a richer and more pluralistic evolutionary theory.

In the last three decades, the explosive growth and explanatory success of evo-devo has begun to accomplish precisely this rapprochement between evolutionary theory and developmental biology (Bonner 1982; Raff and Kaufman 1983; Alberch 1989; Gilbert 2003; Carroll 2008). In the process several traditional, but somewhat vague, ideas about evolutionary and developmental constraints have been successfully sharpened and given a firm footing in the molecular mechanisms of development. In particular the old notion of “internal constraints” on variability (e.g., Whyte 1965), where powerful selection against untenable mutations happens, during development *in ovo* or in utero) can now be more clearly understood in terms of genetic pleiotropy at the level of core developmental mechanisms, and as the result of mutually-constraining interactions within complex genetic networks (see below).

In constructing and testing evolutionary explanations, it is crucial to distinguish the reality of evolution and common descent, which no biologist doubts, from the *importance* of natural selection, which has been a topic of continuing debate since 1859. Unfortunately, this critical distinction between *evolution* (the constrained general process) and *adaptation* (one specific component or result of this process) is frequently disregarded in discussions of human cognitive evolution, and particularly language evolution. Yet the existence of many sources of evolutionary constraint on adaptation (genetic, developmental, allometric and phylogenetic) is a fact, recognized by all practicing evolutionary biologists (cf. Maynard Smith et al. 1985). Natural selection may lead to local optimality, among some set of immediately-available phenotypic options, but *global* optimality is neither required nor expected in evolutionary theory (Jacob 1977; Maynard Smith 1978a).

Darwin himself made his awareness of the distinction between evolution (descent with modification) and adaptation (optimization by natural selection) crystal clear in the Introduction to the *Origin*, writing “I am fully convinced that species are not immutable … Furthermore, I am convinced that natural selection has been the most important, but not the exclusive, means of modification.” (Darwin 1859), using *non*-adaptive traits like “rudimentary organs” and “vestiges” as some of the strongest evidence against the omnipotence of natural selection. Useless traits like the human appendix or external ear muscles, male nipples, rudimentary wings in flightless insects or a rudimentary pelvis in snakes are clearly not adaptations to any current function, but nonetheless have evolved. It was obvious to Darwin that such “vestiges” do not constitute adaptations, but rather are simple reflections of phylogenetic history. Of course, a functional appendix or tail *is* adaptive in many species, but in humans they are simply remnants of an ancient developmental programme. Their very *lack* of usefulness in our own species is what made them such a powerful argument for evolution, and so important for evolutionary theory. Furthermore, Darwin clearly recognized that adaptation often leads to convergent evolution, which may obscure lines of common descent. The streamlined form of dolphins and fish is a convergently-evolved solution to the fluid dynamic problems of swimming rapidly. Their superficial, adaptive similarity should not blind us to the fact that dolphins are mammals, not fish.

Nonetheless, a tendency towards pan-adaptationism has been characteristic of much recent work on cognitive evolution, particularly regarding language evolution, and this has led to a voluminous and increasingly devastating critique of the notion of “adaptation” as it is used in the evolutionary psychology literature (e.g., Gould 1991; Laland and Brown 2002; Buller 2005; Richardson 2007). One recent book enlarges the scope of the critique to all of evolutionary biology (Fodor and Piatelli-Palmarini, 2010). If the entire biological study of cognition—“cognitive biology” hereafter—is to avoid being tarred with the same brush as evolutionary psychology, it is important to address and rebut these charges head on.

Fortunately, as I will show, this is not hard to do. Contemporary biology provides many examples of how to avoid the fallacy that every aspect of every trait is an adaptation (the “Panglossian Paradigm”). Furthermore, the emerging pluralistic explanatory paradigms of evo-devo allow us to clarify the notion of evolutionary constraints in genetic and developmental terms, and thus to more clearly understand the way selection and constraints interact in evolution. Although the application of this more synthetic, pluralistic and comparative approach to human cognitive evolution remains in its infancy, cognitive biologists can learn from exemplary models in functional morphology and the study of the vertebrate brain. The result will be a richer, more biological perspective on cognitive evolution. This viewpoint acknowledges the central importance of natural selection, but treats adaptation, in any particular case, as a hypothesis to be tested rather than assumed. Furthermore, this approach integrates the rapidly-developing understanding of constraints on brain development, and highly conservative genetic and developmental mechanisms, as an important component of evolutionary understanding. My goal here is to show how a balanced pluralistic cognitive biology along these lines is both necessary and desirable (cf. Dor and Jablonka 2010).

### Understanding Constraints: Is the Giraffe’s Neck an Adaptation?

**Fig. 1:**
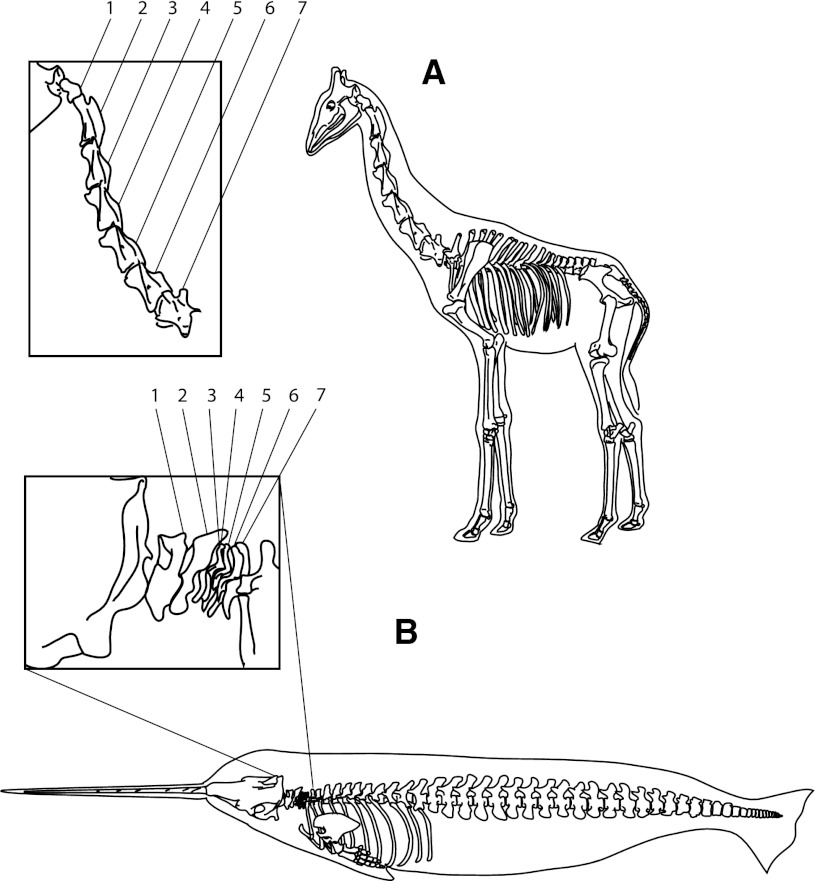
Schematic views of the skeleton of a giraffe and a narwhal, with insets showing that this supremely long-necked species and this virtually neckless cetacean each have seven cervical vertebrae. This is a pan-mammalian constraint, but does not hold in other vertebrates, who may have widely varying numbers of neck vertebrae. Thus, “seven cervical vertebrae” is a nice example of a constraint operating during the evolution of necks in mammals, including humans **a** Giraffe *Giraffa camelopardalis*, **b** Narwhal *Monodon monoceros*

A slightly more difficult situation arises when we attempt to interpret non-adaptive details of an organ which, on the whole, seems adaptive. For example, the long neck of the giraffe has for centuries been considered an adaptation for browsing on high foliage. But giraffes have the same seven cervical vertebrae as other mammals (ignoring definitional sophistry like that of (Solounias 1999)). Indeed, with a very few exceptions, every mammal species (from the neckless whales to the long-necked giraffes or camels) has exactly seven cervical vertebrae (Fig. 1). It would thus be fatuous to attempt to understand the number of bones in a human (or giraffe or whale) neck as an adaptation to bipedalism, browsing, or aquatic lifestyle, and this trait thus provides a nice model system to explore constraints and adaptation in the concrete domain of animal form.

In contrast to mammals, many other vertebrates, like birds or reptiles, have different numbers of cervical vertebrae in long-necked versus short-necked forms (13–25 in birds, p 79: Starck 1979). From an engineering point of view, the option of adding vertebrae during the evolution of long necks is a good strategy: simply repeat a modular structure, adding or subtracting vertebrae until you’ve got an optimal neck length. Indeed, long necks also evolved in some Mesozoic aquatic reptiles like plesiosaurs and elasmosaurs via simple, gradual addition of neck vertebrae (Narita and Kuratani 2005). Thus there is no “in principle” restriction to seven cervical vertebrae among vertebrates as a whole: this is a mammal-specific constraint. This example provides one well-researched example of a developmental constraint interacting with natural selection to produce an end-result with details that are not themselves adaptations (cf. Galis 1999; Narita and Kuratani 2005). It is imprecise to say that the neck of a giraffe is an adaptation to browsing high leaves—we should rather say that giraffe neck *length* is such an adaptation. Other aspects of neck anatomy, such as the number of vertebrae, or the tortuous course of the laryngeal nerve, result from developmental constraints and have not themselves been optimized by natural selection during the evolution of a long neck. Indeed, these constraints appear to have been established long previously, in the first mammals, for they are respected by both monotremes and marsupials, along with virtually all placental mammals (the only exceptions are some sloths, and manatees, Young 1981).

A similar constraint-based logic applies to the human hand: our five fingers may be well-suited to tool manipulation, but they are not *adaptations* for this task. The five-finger default evolved in basal tetrapods long before humans or primates existed (cf. Coates and Clack 1990; Shubin et al. 1997). Any attempt to explain our five fingers as optimal for tool use would be to court the “Panglossian” caricature introduced by (Gould and Lewontin 1979), where the bridges of our noses are seen as adaptations for supporting bifocals. While this seems obvious in these morphological examples, we will see below that the issues are considerably murkier when we come to constraints on neural and cognitive mechanisms, where ancient, phylogenetic constraints play central roles in explaining the final structure and circuitry of the adult brain.

### Where Do Constraints Come From?

There is a long history of discussion of constraints on form, starting with William Bateson’s monumental “*Materials for the study of variation*” (Bateson 1894), and a resulting plethora of terminology for categorizing different types of constraints. I will not attempt to review this literature here. Rather I will provide two exemplary sources of constraints—genetic and developmental—that I think provide a reasonable and uncontroversial starting point for the assimilation of constraint-based thinking to current thinking in cognitive evolution.

### The Genetic Inevitability of Correlated Traits: Pleiotropy and Linkage

Among molecular biologists, genetic drift (the random fluctuation of alleles, particularly important in small, isolated populations) has long been recognized as counter-force to adaptation. Two further well-understood genetic mechanisms underscore the ubiquity of constraints on adaptation. Both result from textbook facts about genetics, and stem from the nature of genes, and their layout (in eukaryotes) on different chromosomes. First, many, probably most, genes play multiple roles in the development of unrelated structures. This multi-functionality is termed “*pleiotropy*” (classic examples are the cystic fibrosis or phenylketonuria alleles in humans). Because of pleiotropy, a new allele arising by mutation will have multiple effects on the organism’s final adult phenotype. If that mutation is, on average, favored in the current environment, natural selection can favor the new allele. Far more commonly, of course, the new allele is dysfunctional on average, and rapidly disappears. Since survival and reproduction is a function of the entire, integrated phenotype of our new mutant, its survival advantage accrues to *all* of the phenotypic effects of the new allele, not just the one or two that may seem obviously relevant to survival. While a new allele of an enzyme may have beneficial effects on digestion, it may have other effects on metabolism, coloration, or other seemingly unrelated factors. Even if these other effects of the gene are, on average, *mal*-adaptive, the new allele may nonetheless increase in the population, if its positive effect on digestion is strong enough to compensate. As a result, it is often said that selection is “blind” to which phenotypic effects are positive and which are negative. Later, of course, further evolution can occur which may moderate or suppress such maladaptive side-effects, but the initial rise of the gene frequency will carry them along inevitably.

A second source of “correlated traits” is *genetic hitchhiking*, (Maynard Smith and Haigh 1974; Hoekstra and Coyne 2007; Williamson et al. 2007; Rubin et al. 2010). Genes are physically located on chromosomes, and the processing of “shuffling” different alleles from one strand to the other is slow, occurring over many generations. Thus, when any particular gene variant is subjected to selection, neighboring regions on the chromosome are also selected in an event termed a “selective sweep”. In the case of powerful selection this neighboring region may be very large, and contain many other alleles that share no functional role with the “target” allele. Such “hitch-hiker” alleles are also selected during a sweep. Later evolution will eventually “break up” these hitchhiking traits from the target trait, but in the meantime considerable allelic variation may have been eliminated from the population, including potentially positive alleles whose importance was outweighed by the target of selection. Because some of the hitchhiking alleles will be present due to genetic drift, particularly in small or isolated populations, the selective sweep “amplifies” the genetic noise that is due to pure chance. Hitchhiking provides another form of correlated variation, distinct from pleiotropy, because hitchhiking effects are due to multiple independent genes, rather than multiple effects of a single gene.

I provide these examples to show how we can understand constraints on evolution in terms of perfectly uncontroversial molecular mechanisms. However, both are relatively weak in the sense that there exist compensatory mechanisms (additional layers of control in the case of pleiotropy, and recombination in the case of hitchhiking) that can overcome them, often in the space of a few hundred generations. Most advocates of the importance of constraints in evolution have focused on a deeper set of constraints, some of which can persist for many millions of years.

### A Menagerie of Constraints

The existence of a more fundamental class of evolutionary constraints on adaptation has long been recognized. Darwin’s colleague and “bulldog” T. H. Huxley acknowledged their importance explicitly (cf. Maynard Smith et al. 1985). Despite this long recognition, they have often seemed a hodge-podge assortment of relatively peripheral phenomena, and various terms have been offered to classify them, including “constraints of growth” (e.g. allometry), “developmental constraints” (e.g. pleiotropy and multiplicity of phenotypic effects), and “phylogenetic constraints” or “phylogenetic inertia” (results of a clade’s “Bauplan”, like the mammal’s seven cervical vertebrae). Most biologists today take it for granted that the complexities of development and the vagaries of phylogenetic history tightly constrain many adaptive processes. Note that the term “constraint” is a bit misleading, because historical and developmental forces don’t simply limit adaptation, they also bias and channel the generation of phenotypic variation: they provide the scaffolding upon which natural selection acts (cf. Kirschner and Gerhart 1998, 2005).

*Developmental constraints* have been defined by Maynard-Smith and colleagues as “biases on the production of variant phenotypes, or limitations on phenotypic variability, caused by the structure, character, composition, or dynamics of the developmental system” (p. 265, Maynard Smith et al. 1985). In general, fundamental genetic bases of development are extremely conserved, and in many cases shared by all living metazoans. Thus evo-devo embraces genetic and phylogenetic constraints under one broad umbrella: the conservation of developmental mechanisms (Gilbert et al. 1996; Kirschner and Gerhart 2005; Carroll 2008). Because they are ubiquitous, developmental constraints probably play the most important role in the overall effect of constraint on the evolution of complex traits. For any organ, we expect certain details of form and physiology to represent adaptations, while others reflect phylogenetically conservative developmental constraints of various sorts, or spandrels (Gould and Lewontin 1979). Influenced by evo-devo, recent overviews of human anatomy have emphasized such historical constraints on human biology (Shubin 2008; Held 2009).

Gould, a champion of the importance of constraints in evolution throughout his career, offered two other examples of constraints. First, “constraints of growth” are effects on details of the overall phenotype caused by selection for specific traits (Gould 1977); when the selection is on body size, the changes in shape that co-occur are studied under the rubric *allometry*. There is a long history of allometric study (Huxley 1932; Thompson 1948; Gould 1966; Finlay et al. 2001), but its developmental basis remains unclear. Second, a trait that arises as a spandrel may be later, opportunistically, be put to use, and Gould argued that such exaptations are particularly relevant to cognitive evolution (Gould 1991).

### A Constraint Explained by Evo-Devo

To illustrate, let us return to the giraffe’s neck. The last decades have brought important breakthroughs in our understanding of the developmental system patterning the spinal column in mammals and other vertebrates, allowing us to clarify traditional notions like “Bauplan” in mechanistic, developmental terms. Briefly, vertebrae are formed in the embryo from somitomeres, which are repeated structures running down the embryo’s back. The process of somitomere formation—somitogenesis—involves oscillatory changes in gene expression as the embryo grows (Pourquié 2003; Lewis 2008). During development, an oscillatory process in time (the “segmentation clock”) is converted to a repeated pattern of vertebrae in space (the future spinal column). This system is reminiscent of traditional models in mathematical biology (e.g., Turing 1952; Wolpert 1969), first proposed as a concrete hypothesis much later, by Lewis (1978). Today, we know that many genes, engaged in interacting oscillatory “circuits”, are involved in somitogenesis (Aulehla and Pourquié 2008; Gomez et al. 2008). This is thus an excellent arena to ask how new findings in genetics and development can illuminate old ideas about the mathematics of pattern formation, and even older ideas about the vertebrate Bauplan and the role of constraints in evolution.

A core principle of evo-devo is that, although phenotypes may vary widely among living organisms, the underlying developmental processes are often the same, even to the point of utilizing identical genes in analogous ways (Gilbert 2003; Carroll 2008). The genetic and developmental toolkit remains constant even when the output of the developmental “workshop” varies considerably between species. Thus, the process of somitogenesis is shared among all vertebrates, and indeed many of the underlying genetic mechanisms are also used to build the body segments of insects and other arthopods. This is an example of “deep homology”, the surprising finding of identical genetic mechanisms underlying similar traits in widely-divergent taxa like giraffes, frogs and fruit flies (Shubin et al. 1997; Carroll 2008; Shubin et al. 2009). It also is an example of a deep developmental constraint on evolution: despite some flexibility of somitogenesis across taxa, tampering with this system can lead to major, and typically fatal, changes in any particular embryo.

**Fig. 2:**
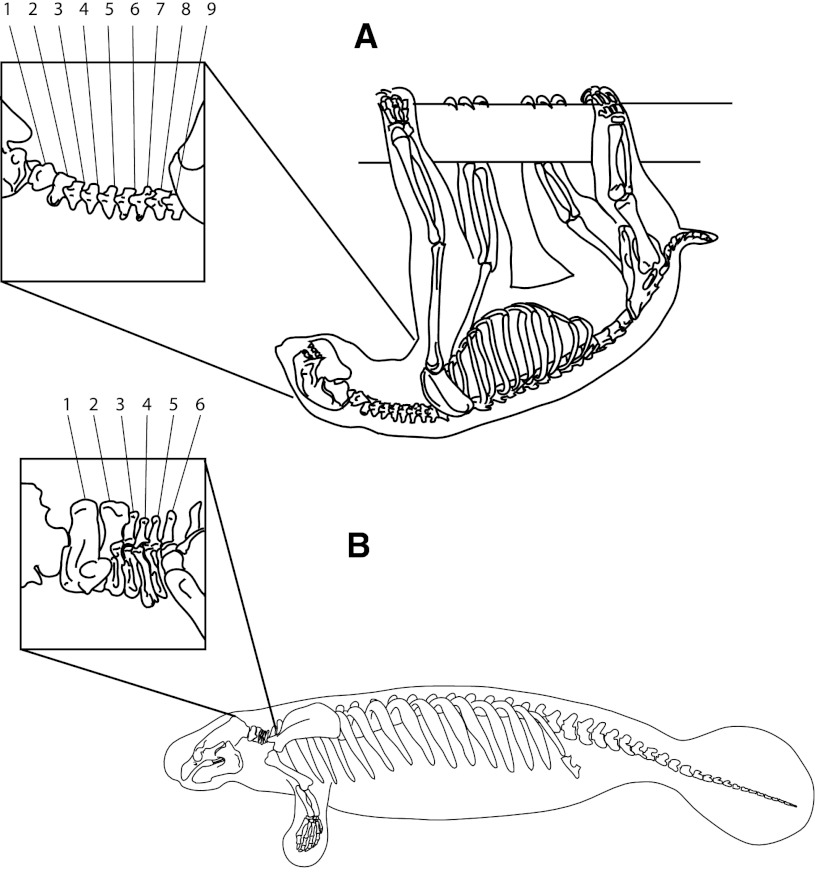
*Mammalian Exceptions*: The only mammals to have fewer or more cervical vertebrae than the seven typifying all other mammals are manatees (genus *Trichecus*) and sloths (genera *Bradypus* and *Choloepus*). Long necked sloths are actually “exceptions that prove the rule”: what appear to be extra cervical vertebrae are actually just thoracic vertebrae that have lost their ribs. The situation in manatees and short-necked sloths remains a topic of current investigation. **a** Sloth *Bradypus tridactylus*, **b** Manatee *Trichecus manatus*

Thus, giraffes and other mammals have seven neck vertebrae because the entire spine is patterned by an ancient developmental system, and changing this system has implications for many aspects of morphology (not just neck length). A mutation which changes the timing of somitogenic gene expression, or the interactions between genes in the segmentation clock, can have multiple, often drastic effects (Ishimatsu et al. 2010). These linked changes often lead to a failure during development. Embryonic death represents a form of “internal selection” during development (Whyte 1965; Raff 1996). Although it remains unclear why mammals are more constrained by this system than other vertebrates, considering the apparent exceptions to the rule provides some clues. The only mammalian exceptions to the “rule of seven” are sloths (with six to ten neck vertebrae) and manatees (with six) (see Fig. 2). However, it now appears that the “extra” cervical vertebrae in *Bradypus* sloths are simply thoracic vertebrae that have lost their ribs (Bell 1834; Hautier et al. 2010), and actually are an exception that proves the rule. The situation in manatees and short-necked *Choloepus* sloths is less clear, but in both cases the cervical changes seems to reflect more global changes in overall spinal patterning, so that the change in cervical count is a by-product of some overall adaptive change (Buchholtz et al. 2007; Buchholtz and Stepien 2009). These aspects of mammalian spinal patterning may be more “locked in” by a system of mutually-constraining, coupled gene circuits and/or developmental mechanisms, than in other vertebrates like birds (Galis 1999). This hypothesis is supported by the finding that changes in neck vertebra count *can* be found in humans, and are indeed relatively common: but only in spontaneously aborted embryos, or dead infants (Galis et al. 2006). This is a perfect, if chilling, illustration of the importance of “internal selection” in development, and its role in the evolution of the human body.

Developmental constraints thus have profound effects on morphology, and can be specified in mechanistic terms in a way relevant to understanding the evolution of form and function. The new perspective of evo-devo allows us to clarify one of Darwin’s central themes—descent with modification—in modern terms. We can thus better understand many aspects of the phenotype as resulting from phylogenetic and developmental constraints, which ultimately result from historical factors, not adaptation. But this perspective also dispenses with any appearance of conflict between adaptation and constraint: both are central components of any full explanation of a trait. Constraints provide the context in which adaptation occurs, by filtering many non-functional phenotypes long before they are born and exposed to environmental selection.

### Distinguishing Adaptive Traits from Non-adaptive Byproducts of Constraints

The existence of correlated traits, whether stemming from developmental or genetic constraints, leads to a quandary. Biologists are interested not only in the history of a trait, but also in the causal forces which lie behind this history. As already shown, we need to accept the existence of correlated traits which do not, in any direct way, have a current causal role in increasing survival and reproduction. Thus, we need some way of distinguishing a “target” trait of natural selection, the variant that leads to increased survival/reproduction, from the various correlated traits that typically come along for the ride. This is a core problem that the existence of constraints and correlated traits forces us to confront. It is particularly important when we consider the details of cognitive traits, where the fine-tuning of neural circuits during development may incorporate underlying constraints into the final circuit. The robustness of development leads to circuitry which is functional and seems adaptive, but which is not in fact an adaptation in the strict sense of (Williams 1966a).

Distinguishing correlated traits from those specifically selected is not a trivial problem, in two distinct senses. First, it has no general solution: we need to solve it on a case-by-case basis, depending on the trait, its genetic basis, the phylogeny of the species, and the relevant ecological and selective circumstances. Thus, at a minimum, we need to know a lot about an organisms’ physiology, phylogeny and ecology before we can even hope to distinguish targeted from correlated traits.

Second, if we were to fail, in general, to distinguish them, the explanatory power of the theory of natural selection is called into question. In particular, if we are to save evolutionary theory from being tautological (“selection is survival of the fittest, and the fittest are defined as those who survive”), we need some independent way of distinguishing the trait(s) “selected-for” versus those that are merely “selected”. This quandary has received surprisingly little attention from philosophers of biology (though see Sober 1993; Gould 2002), but has recently been brought to a head by critics of evolutionary psychology (e.g., Buller 2005). In particular, a recent sweeping attack on the entire theory of natural selection is based on precisely this problem (Fodor and Piatelli-Palmarini 2010). Fortunately, although the problem is real, we need not accept the policy of despair offered by these authors, because practicing biologists have had a working solution for many years.

The theory of natural selection holds that animals vary, that offspring resemble their parents, and that not all organisms survive and reproduce equally. If any inheritable variation is causally related to survival, those with the advantageous variation will, on average, survive longer, reproduce more, and increase in the population. This much is clear and true, and is clearly not tautological. The problems arise when we attempt to anatomize the successful variants produced by natural selection, singling out some anatomical, physiological or behavioral trait as “an adaptation”. Given the ubiquity of correlated variation, we want some non-arbitrary way of determining which aspects of the variant trait are, and which aren’t, causally related to increased survival and reproduction. Neither Darwin’s theory, nor modern neo-Darwinism, offers any automatic algorithm or simple definitional criteria for distinguishing such adaptations from mere correlated traits. Nonetheless contemporary biology provides many clear examples of how to make the distinction, via a process of hypothesis-testing, contingent upon the trait and species in question. This scientific discrimination process is intrinsically post hoc in the usual (and not pejorative) sense that all discussions of biological history must be: we have data about the current situation (and, if we are lucky, a fossil or two) and we attempt to use these data to understand past historical processes that led to the current situation.

There are two prime tools at our disposal. The first is *comparative*, and involves the reconstruction of phylogenetic history based on existing species. We first examine the trait in question in many related species. If we find *homologies* in related forms, we use them to deduce the evolutionary time period during which the variant evolved. Applying this technique to the giraffe’s neck, we find that the giraffe’s closest living relative, the okapi, has a somewhat elongated but relatively normal neck, as do most of giraffe’s more distant relatives among hoofed mammals. Even without examining the fossil record, we can clearly state that an unusually long neck is a derived trait of giraffes. In sharp contrast, counting neck vertebrae, or many other anatomical and physiological features of the giraffe’s neck, we find characters that are widely shared not only with okapis but with virtually all mammals. None of those are good candidates for “giraffe-specific adaptations”. Indeed, if they vary so little in the large clade of all mammals, they are excellent candidates for *constraints* on adaptation.

The comparative approach does not stop there. Crucially, if comparative research uncovers examples of *convergent* evolution, we can use them to test evolutionary hypotheses and derive other conclusions. Convergence may help us discern the function of the trait more clearly, by observing similarities and differences in species behavior or ecology. Convergence may also help us to distinguish between the adaptive and non-adaptive components of the trait (e.g. isolating the lens and iris as presumably adaptive components of vertebrate and cephalopod eyes, and the inverted vertebrate retina as presumably non-adaptive: Walls 1942; Allman 1999). Finally, if a well-defined trait has evolved convergently often enough, we can use such examples as independent data points to empirically test our hypotheses about function. This final point makes analysis of convergence a core component of the modern comparative method, using independent contrasts (cf. Harvey and Pagel 1991). Thus a broad comparative and phylogenetic approach is one source of evidence bearing upon both specific adaptations, and constraints upon them.

The second tool involves adopting an *engineering perspective*, in which we detail the (hypothetical) adaptive problem or problems “solved” by some characteristic, and then use principles of physics and physiology to make *predictions* about how the trait should vary. Such an engineering approach is what allows biologists to evade the charge of circularity in definitions of fitness and adaptation. The fittest are not simply those who survived, but rather those whose survival was causally linked to their possession of some trait, and where specific components of that trait can be causally linked to the solution of some problem habitually faced by the species in question. Depending on whether the problem in question is digesting some novel sugar, efficiently finding food, running faster than predators, or simply hiding from them via camouflage, we expect quite different engineering principles and constraints to be relevant.

This approach is termed “reverse engineering”, sometimes disparagingly (e.g. Buller 2005), but involves nothing more than scientists tackling a difficult problem by using all of the information available. The methods adopted are often those of engineering design, including especially abstraction/idealization of the problem, and then use of optimization theory to find the best solution given various specific constraints (Maynard Smith 1978a). Different hypotheses about the adaptation invoke different principles, and imply different optimal solutions. If wing feathers are an adaptation for heat retention and thermal insulation, we expect certain characteristics. If they are adapted to powered flight, we predict others. Having made such predictions, we return to our feather collection and examine, or test them for the predicted characteristics (cf. Gould and Vrba 1982). In this case, we find that wing feathers in living birds bear traits (e.g. asymmetry) that are explicable based on optimization for powered flight, but not as insulation. The same examination leads to the opposite conclusion for nestling feathers or for adult down, which in fact *are* optimized for heat retention.

In principle, the combination of comparative and engineering approaches allow us, through a process of empirical hypothesis testing, to tease out what aspects of a trait are adaptive, and what problem(s) they are adaptations “for”. This two-pronged approach provides a ready answer to the criticism of tautology (Fodor and Piatelli-Palmarini 2010): we use extra-evolutionary principles to cash out our predictions about the function of and selective forces that acted on the trait of interest. Both aspects of this approach were familiar to Darwin, and both are ubiquitous in modern biology. While Fodor and Piatelli-Palmarini are correct that neither Darwin nor modern evolutionary theory provide us with any sweeping “laws of selection” of uniform applicability to all traits and all species, this is hardly a worrying criticism. It is like criticizing Newton’s laws for not providing all relevant details of contemporary fluid dynamics needed to build efficient submarines and airplanes. No general theory can specify all such particulars. Indeed, the invocation of particular, problem-specific engineering principles of physics and physiology is *precisely* what allows evolutionary theory to escape the charge of tautology. Although Dobzhansky was right that nothing in biology makes sense except in the light of evolution (Dobzhansky 1973), it is equally true that nothing in evolution makes sense except in the light of biology more generally (including its mechanistic grounding in the other natural sciences).

Unfortunately, the degree to which we understand the relevant physics and physiology varies considerably from trait to trait. Physicists and engineers know a lot about aerodynamics, and thus can say a lot about the functional morphology of bird, bat and insect flight (Dickinson et al. 2000) or the mechanics of chewing, digging, or vocalization (Hiiemäe and Ardran 1968; Biewener et al. 1985; Alexander 1992; Pough et al. 1996; Fitch and Hauser 2002). The clearest examples thus come from these domains of “functional morphology”. When we turn to cognition and neural computation, the relevant engineering principles are much less clear, posing major unsolved problems for those interested in the evolution of behavior and cognition.

## Part Two: Distinguishing Constraint From Adaptation in Human Evolution

This discussion leads me to the following proposal: constraint-based thinking in models of language evolution requires recognition that many aspects of modern human language do not, in any meaningful sense, constitute adaptations. Rather, they reflect deep developmental and phylogenetic constraints on the physiological and neural mechanisms underlying linguistic behaviour. Furthermore, the multiple functions that language performs in modern humans make it a challenge to highlight any one of them, such as communication or thought, as “the” central adaptive function of language, at present or in the evolutionary past. Finally, the fact that languages are learned—a form of phenotypic plasticity—complicates the explanatory situation considerably. When we find some apparently optimal situation in adult language, it is always possible that this optimality results from fine-tuning during ontogeny, rather than adaptation during phylogeny. A reverse engineering approach will thus have only limited success until it is paired with a deeper understanding of the constraints on cognitive and neural evolution, and on neural plasticity. Because this argument remains controversial, and even inflammatory in certain circles (cf. Fodor and Piatelli-Palmarini 2010; Botha 2011), I will support my contention using two examples: the evolution of bipedalism, and the evolution of the human vocal tract.

### Constraints, Adaptation and the Evolution of Bipedalism

Bipedalism refers to terrestrial locomotion on two legs (running, walking, hopping, etc.). Much is known about how human walking and running work mechanistically. A good hominin fossil record of hips and hindlimbs, and hominid footprints at Laetoli (Leakey and Hay 1979), makes bipedalism one of the best understood aspects of recent human evolution. Despite this knowledge, the adaptive *function* of human bipedalism remains obscure, and functional morphologists often dismiss discussions of the adaptive origins of bipedalism as speculative story-telling (e.g.,Hutchinson and Gatesy 2001).

Unlike language, bipedalism has evolved repeatedly among other animals. The mechanics of walking and running have been well studied in both humans and birds, and rest upon similar mechanical principles, allowing “reverse engineering” (Dickinson et al. 2000). Birds provide the most numerous and obvious example, but apes and monkeys can walk bipedally for short periods, and various lizards or insects sometimes run bipedally. Although unique in some details (Alexander 2004), many details connected with human bipedalism are also observed in such species (cf. Gatesy and Biewener 1991; Hirasaki et al. 2004). These multiple convergent exemplars of bipedal locomotion help us test hypotheses concerning the evolution of human bipedalism. For example, a key convergent parallel is the bringing of the feet into line nearly directly beneath the midline. When lost, as in short-legged penguins (Griffin and Kram 2000), the result is a shuffling inefficient walk. This change from the ancestral “sprawling,” vertebrate gait is one key to greater bipedal efficiency. Such comparative data also illustrate the requirement for specific anatomical changes to support efficient walking.

Turning to the *adaptive* function of walking, we find an obscure situation. Numerous hypotheses (at least 30) have been offered concerning why humans became bipedal in the first place (reviewed in Niemitz 2010). The old idea that bipedalism was a reaction to tool use, to allow more efficient carrying, is disproved by the fact that sophisticated stone tools followed bipedalism in the fossil record by several million years. The current “standard” hypothesis is that the energetic advantages of bipedal walking drove its evolution. Other widely-discussed possibilities include a decrease in the surface area of the body exposed to solar radiation, raising the head above grass and obstacles for a better view, more impressive displays during conflicts, or adaptation to aquatic foraging. That bipedalism is *beneficial* in these ways seems clear, but proposals that bipedalism is a biological *adaptation* to any one (or several) of them have proved very controversial indeed.

Furthermore, the energetics of human walking and running differ considerably (Taylor et al. 1970; Taylor and Rowntree 1973; Rodman and McHenry 1980): bipedal walking is slightly more efficient, and running considerably less efficient, energetically speaking, than the corresponding quadrupedal gaits. The efficiency of human walking rests on an “inverted pendulum” mechanism: in mid-stride, the center of gravity is at its highest point, and stores energy that is released during falling toward the end of the stride. Adaptive explanation for bipedalism must explain not only the value of walking, but also why the costs of bipedal running did not outweigh those advantages. One possibility is that human endurance running is unusually flexible in tempo, due to a breaking of constraint tying respiration to footfalls. In *Homo*, this flexibility might have proved advantageous in sustained chasing of game, despite increased energetic costs (Bramble and Lieberman 2004).

Clearly, even for this relatively simple and mechanistically well-understood change in human form, with a good fossil record, the question of adaptive function is fraught with ambiguity and controversy. One potential solution is to be more specific about what, precisely, we aim to explain with particular adaptive hypotheses and admit that “Bipedalism” as a monolithic entity is too broad to allow a single, simple causal explanation. Bipedal walking and bipedal running may have different historical origins and functional causes, separated by long time periods. For example, human legs have long, spring-like Achilles tendons which store and release energy during running. These are absent in apes, and serve little purpose during the “inverted pendulum” of human walking. It is thus sensible to ask whether compliant Achilles tendons, or similar details like toe shortening, are an adaptation to *running* (Bramble and Lieberman 2004). At even a finer level, we can ask whether breathing through the mouth (typical in human runners) versus the nose (habitual in apes), which Bramble and Lieberman offer as a potential “key innovation” during human evolution, is an adaptation to *endurance* running.

Let us turn now to constraints. One problem in considering bipedalism from an adaptive viewpoint is that many historical, developmental constraints clearly influence the evolution of body form and thus constrain locomotory evolution (Maynard Smith 1978b). Bipedalism evolved within a context of morphological constraints like the number of leg bones and physiological constraints of bone strength, muscle properties, the rhythm of breathing, and neural control of balance that long predated bipedalism. Such details are central to understanding adaptive function in locomotion in any species, and an optimization analysis starts with an understanding them, and how they restrict the range of developmental possibilities (Maynard Smith 1978a).

To be “possible” a variant must be able to survive developmentally (unlike, for example, modifications in neck vertebrae number (Galis et al. 2006)) and also survive tradeoffs caused by use for multiple purposes (e.g. between efficient walking *versus* running). There can be little doubt that many of the medical difficulties that human suffer (bad knees, back problems, hip replacements, problematic childbirth…) are the direct result of the novel and recent exaptation for bipedalism of an ancestral limb structure adapted to quadrupedalism (Held 2009).

Finally, not all aspects of morphology that are “adaptive” in the ordinary sense of working efficiently are “adaptations” in the evolutionary sense. Considerable research on bipedalism in normally quadrupedal mammals, including monkeys, rats and goats, shows that animals forced to locomote on their hind legs can do so. When bipedalism is enforced, from a young age, multiple aspects of skeletal anatomy in the skull, spine and pelvis, change developmentally to resemble the anatomy of humans (e.g. a curved spine or splayed pelvis) (Slijper 1942; Moss 1961; Kay and Condon 1987; Hayama et al. 1992; Nakatsukasa et al. 1995). Without a solid understanding of such phenotypic plasticity, it is quite difficult to separate mutation-driven, inheritable changes which might constitute adaptations from ontogenetic accommodation to bipedal *behaviour* in our ancestors.

To summarize, human bipedalism is well-studied and in many ways well-understood. But despite these virtues, questions regarding the adaptive function(s) of bipedalism are mired in controversy. This offers a clear lesson regarding language evolution, since we are far more fortunate regarding human bipedalism than for human language. Bipedalism illustrates the need to focus on mechanisms and developmental constraints and to sharpen our questions about what details we hope to explain as adaptations, if we are to have any hope whatsoever of understanding the phylogenetic history and adaptive functions of human language.

### The Evolution of Speech: Human Vocal Tract Anatomy and Vocal Imitation

A second example of the interaction between adaptation, exaptation and constraints is provided by the evolution of human vocal tract anatomy in humans (cf. Fitch 2000, 2010). Adult humans have a low-lying larynx compared to most other mammals: the larynx and hyoid bone (which anchors the tongue base) are retracted caudally (downwards). As already noted by Darwin, this lowered larynx appears to increase our risk of choking (Darwin 1859). For decades this bizarre conformation was believed to be uniquely human, and an obvious adaptation to speech, because the associated change in tongue shape allows humans to make vocal tract shapes thought impossible with a “normal” high larynx (Lieberman and Crelin 1971; Lieberman 1984). The status of the descended larynx as an adaptation for speech went unquestioned for many years (Lieberman 1975, 1984; Pinker and Bloom 1990; Lieberman 2000; Pinker and Jackendoff 2005).

In 2001 my colleague David Reby and I were surprised to discover that some deer species have permanently descended larynges (Fitch and Reby 2001). Since then, a similar, permanently-retracted larynx has been found in other mammals, including several gazelles and all of the big cats (Weissengruber et al. 2002; Frey and Riede 2003). Since none of these nonhuman species produce speechlike vocalizations, these findings raised an obvious question: what non-speech function might a descended larynx serve? Fortunately, earlier work had already clarified a plausible alternative hypothesis: that a retracted larynx serves to elongate the vocal tract, leading to lowered formant frequencies that convey an acoustic impression of increased size (Fitch 1994, 1997). The simple but very impressive roars of these nonhuman species was consistent with this “*size exaggeration*” hypothesis. Considerable further work has confirmed key components of this proposal: formants often correlate with body size (e.g., Fitch 1997; Reby and McComb 2003; Riede and Titze 2008), and humans, dogs, and deer use this information when judging body size (Smith et al. 2005; Charlton et al. 2008; Taylor et al. 2011). Furthermore, a secondary descent of the larynx occurs in humans at puberty (Fitch and Giedd 1999; Lieberman et al. 2001), but only in males. This second descent lowers formants without increasing speech abilities, allowing the size exaggeration hypothesis to also account for this previously unexplained aspect of human vocal tract anatomy (Fitch 2002).

With these new findings in hand, we may ask whether human vocal anatomy is an adaptation for language, specifically for vowel production. Certainly, we humans *use* our reconfigured vocal apparatus to produce distinctive vowels, and the world’s languages have developed vowel systems exploit this capability (Liljencrants and Lindblom 1972; de Boer 2001; Oudeyer 2005). But usefulness does not demonstrate adaptation for speech, and even today, the “extra” laryngeal descent that occurs during puberty in males alone does *not* appear helpful for speech, and in general women’s speech abilities are superior to men’s (Maccoby and Jacklin 1974; Henton 1992). Male-specific pubertal descent thus seems unlikely to be an adaptation for speech, and more likely, is an adaptation for size exaggeration, just as in deer or lions (Fitch 2002).

An additional argument has occasionally been made, that the human larynx descends for purely mechanical reasons due to the assumption of upright posture and a change in skull conformation. By this argument, the descended larynx would be a spandrel—a necessary byproduct of other, adaptive changes—and the undoubted usefulness of this trait for speech is just like the usefulness for the bridge of our nose for supporting sunglasses.

A second possibility is that the descended larynx initially evolved as an adaptation for size exaggeration, was later *exapted* for phonetic use, and that subsequent evolution has converted it to a true adaptation for spoken language. By this argument, the descent of the larynx *which occurs during infancy in both sexes* can plausibly be hypothesized to be an adaptation for the production of vowels, as originally hypothesized by Lieberman and colleagues, while pubertal adult male descent remains an adaptation for body size exaggeration. Currently, no one has done more than make a plausible case (e.g., Lieberman 1984) that this anatomical change improves communication, and thus increased the survival and reproduction of ancestral males who possessed it.

Turning briefly to a different aspect of the evolution of human speech, our capacity for *vocal imitation of complex sounds* is unique among primates, but shared with other vertebrates including many birds (Nottebohm 1975; 1976; Marler 2000) and some mammals (Janik and Slater 1997; Fitch 2000). Vocal imitation results from changes in neural control, not vocal anatomy, as illustrated by talking seals which have a high resting larynx position but, due to their neural capacity for vocal learning, can imitate speech (Ralls et al. 1985; Deacon 1997). Much has been learned about vocal imitation in recent years, and its convergent evolution in other species provides an excellent opportunity to test ideas about its development and evolution in humans (cf. Doupe and Kuhl 1999; Jarvis 2007; Matsunaga and Okanoya 2009; Fitch 2011a). Is vocal imitation an “adaptation for speech”?

Vocal imitation is key to both speech and song in humans, and is shared with other species who use it in vocalizations termed “song”. One hypothesis about the evolution of speech posits an intermediate state, a “musical protolanguage” more like modern song than speech (Darwin 1871; Mithen 2005; Fitch 2006). Darwin cited vocal imitation in birds as clear evidence that selection for song *can* drive the evolution of vocal imitation, and more recent discoveries strengthen that argument: virtually all non-human vocal imitators use the skill in songlike behaviour, and none produce anything resembling speech or spoken language. Accepting the basic plausibility of this hypothesis, questions about whether vocal imitation first evolved for song-like purposes or speech-like purposes will remain difficult to resolve. Was song the original function of vocal imitation (Darwin 1871), with speech an exapted byproduct? Or is song simply a spandrel of speech, of no adaptive value (Pinker 1997)? Again, despite significant advances in understanding the *mechanistic* basis of speech, this historical *adaptive* question will remain challenging. In conclusion no confident assertions about the adaptive value, for speech, of a descended larynx or vocal imitation in humans (e.g., Lieberman 1984; Pinker and Jackendoff 2005; Lieberman 2007) can be justified by currently available data.

## Part Three: Adaptation and Constraint in Human Cognitive Evolution

So far, I showed that phylogenetic and developmental constraints are ubiquitous and inevitable, and I argued that constraints must play a central role in explaining and interpreting organismic form and function. I described how we can distinguish the properties of an organism that result from historical constraints (e.g. pleiotropy, allometry, unmodified “spandrels”) *versus* those that constitute adaptations, by using a reverse engineering approach and comparative tests on convergently-evolved traits in multiple clades. Though this empirical distinction is both necessary in principle, and occasionally accomplished in practice, it requires laborious, time-consuming scientific work. As a result, even in well-researched examples like bipedalism and speech, many core questions about adaptive function remain completely unresolved, and are likely to remain so. Adaptive hypotheses may serve as useful “intuition pumps” to drive empirical research, helping to provide a source of plausible hypotheses to be tested, and I provide three such hypotheses elsewhere (Fitch 2011a). But unless treated skeptically, tested and thoroughly integrated with empirical, comparative research, adaptive hypothesis generation runs the risk of falling prey to the Panglossian paradigm caricatured by (Gould and Lewontin 1979).

In the rest of this paper, I will argue that this risk is particularly great when considering the evolution of the brain and cognition (Gould 1991; Buller 2005; Richardson 2007). The problem, which is bad enough for morphology, is compounded by rampant phenotypic plasticity in the brain. Animal nervous systems are designed for flexibility and learning. Individuals can achieve novel phenotypic states that have no direct evolutionary precursors (e.g. piloting airplanes or playing chess or the cello), building upon more general, and often ancient, capacities for perceptual learning or motor control. Of course, this simple fact does not imply that there are no evolutionary precursors relevant to understanding brain function in jet pilots and grand masters. There are a host of extremely relevant factors, but *all* of them belong in the category of pre-adaptive constraints *vis a vis* airplanes and chess. They are based on what Andrews and colleagues aptly termed “exapted learning mechanisms”, not specifically designed for piloting or chess (Andrews et al. 2002). Understanding the structure of such systems requires a thorough understanding of the neural constraints on skill acquisition, and the genetic constraints on brain development, and no appeal to the adaptive function of chess is necessary or justified.

I presume that the above statement about the primacy of constraints over adaptation for chess or airplane flight is uncontroversial. I suggest that this primacy is equally relevant for most other aspects of human cognition, particularly including language, because its evolutionary history is so short. Many aspects of human spoken language thus cannot be properly viewed as adaptations to their current function in language. Instead, they reflect far more ancient phylogenetic and developmental constraints on cognition and learning mechanisms, some of them perhaps “ghosts of adaptations past” broadly shared with many other animals. This conclusion, if correct, is bad news for the traditional evolutionary psychology approach, which seeks the phylogenetic history of human cognition finetuned to a Pleistocene “environment of evolutionary adaptedness,” or EEA. Rather, the history of evolutionary adaptation for human cognition must extend back to the Cambrian, and right through to the present. As Darwin suggested in the quote above, we should be prepared for a series of adaptations for different functions over our long evolutionary history from fish to primates. By this model, the human brain will present a palimpsest of pre-adaptation, exaptation and re-use of old parts for new purposes.

### Adaptation and Constraint in the Brain

When we attempt to distinguish adaptations from constraints, the nervous system provides some particularly interesting, and difficult, problems. Organisms can learn, and the evolved capabilities of any particular species to do so are constrained by many biological factors. Even organisms with simple nervous systems have some capacity to adjust to current circumstances and learn (Walters et al. 1981). This capacity for conditioning is, however, tightly constrained by prepotent innate response capabilities (Levy and Susswein 1999). For example, rats can learn to associate sounds with shocks, and tastes with nausea, but not vice versa (Garcia and Koelling 1966). Indeed, outside of the Skinner box, most organisms have very clear biases on what they can easily learn, and when (Breland and Breland 1961). These biases, often grounded in reliably-developing species-typical behavior (hereafter termed “instinct”) are central to our understanding of the evolution of learning and cognition.

To what degree do constraints and biases on behaviour, and proclivities for learning, evolve rapidly, to be honed to recent adaptive demands (as evolutionary psychologists argue)? To what degree is the human instinct to learn language an adaptation for language *per se*, rather than motor skill learning, culture acquisition or general cognitive ability? I believe that human cognitive and linguistic capabilities rest, for the most part, on an ancient shared basis, and thus that the role of phylogenetic and developmental constraints has been drastically underestimated in much of the recent work on language evolution, and human cognition more generally.

My argument rests on the fact that many ancient adaptations exist in the vertebrate nervous system that allow it to “wire itself” in ways that are highly functional for the individual, but are not adaptations of its species. The neural circuits that result from this self-wiring process may be very specific to aspects of language (speech production or perception, syntax parsing, semantic processing, or even reading or writing) without themselves being adaptations for these tasks. I suggest that many components of modern language processing fall into this category, and thus that many of the constraints on human languages actually result from ancient neural and cognitive constraints that preceded language, evolutionarily. Additionally, those aspects of human cognition that *are* unique to our species, and that evolved as recent adaptations, may play an important role in non-linguistic cognition as well, such as social cognition, music or tool-making. To the extent that these non-linguistic behaviors played a selective role on the evolution of that ability, it is also misleading to term them “adaptations for language”. Unfortunately, as is already clear from the examples above, there is little hope, with current techniques, of uncovering the facts of the matter about such past selective forces.

The bleak outlook for understanding past selection stands in sharp contrast to our ever-increasing power to understand developmental mechanisms and phylogenetic constraints from a comparative, empirical viewpoint. Thus, to the extent that these arguments are correct, we should abandon fruitless arguments about whether “language is an adaptation” (Fitch et al. 2005; Pinker and Jackendoff 2005). We should replace them with data-driven discussions about the specific mechanisms that allow language processing, and with empirical investigations of the constraints on those mechanisms.

### Cognitive “Adaptations”: Evolutionary and Ontogenetic Sources

Comparative work on a diversity of animal species will be crucial for answering the latter two questions. Above, we saw that quadrupeds forced to walk on their hind limbs from a young age develop skeletal characteristics similar to those of humans epigenetically. These clearly represent developmental adjustments to unusual circumstances. While “adaptive” in the normal English sense of helping the animals to walk bipedally, such ontogenetic changes are not adaptations to bipedalism in the Darwinian sense. Of course, the developmental flexibility allowing bones to grow in various appropriate ways, depending on developmental circumstances, is adaptive. But we must clearly distinguish the evolutionary process by which *this* general functional process was achieved, from the phenotypic outcome it led to during bipedal ontogeny. If we are to designate *that* generative, developmental process an adaptation, we must also specify what it is an adaptation *for,* and this is where the problems begin. Without a detailed historical account of how such a flexible system evolved, and the reasons that flexible organisms prevailed over less flexible ones, we will be unable to do so.

Might developmental flexibility always be favored? Is flexibility simply a goal towards which evolution always strives? This seems unlikely: some species are more flexible than others, and specialist species often evolve from generalists. The elaborate specialized beaks of Darwin’s finches provide a nice example (Grant 1986), as does the history of mammalian evolution in the Tertiary, with anteaters, sloths, rhinos and dolphins evolving from an ancestral mammal that was a rat-like generalist (Simpson 1949; Ji et al. 2002). Often, it seems, specialization is favored over generalism, and we cannot assume generalists (or generalist brain development) as a default.

Flexibility in nervous system development also varies considerably across clades. The nervous system of many invertebrates develops in a pre-determined fashion, with identifiable neurons, essentially identical across individuals, playing the same role, making the same connections, and expressing the same genes. Such fixed, identified neurons do not exist in the human brain, or in vertebrate brains in general. The vertebrate brain develops through a more flexible and interactive epigenetic process, typified by exuberant growth and overproduction followed by experience- and situation-dependent pruning (Purves and Lichtman 1980; Brown et al. 1991). This neural developmental process has been evocatively termed “neural Darwinism” (Edelman 1987), and constitutes a more flexible alternative to the pre-determined development typifying some invertebrate clades. Although neural epigenesis shares certain characteristics with the evolutionary process, they obviously must be distinguished: evolution is about changes in whole populations, while ontogeny involves changes in an individual. We expect nervous development, at least in vertebrates, to produce many circuits that are well-tuned to the individual organism’s environment, but we do not expect each of these to be an adaptation in the Darwinian sense.

Consider frog vision. Frogs have essentially separate visual fields for each eye, with a fully crossed optic chiasm and no overlap between the two retinal projections. In contrast, in cats, monkeys, or humans, who have a large binocular overlap, the visual projections arrange the contribution of each eye into adjacent alternating “dominance columns” from the left and right eyes. Frogs normally show no such striping, because each optic tectum has projections from one eye alone. But if a third eye is experimentally grafted onto a tadpole’s head, during metamorphosis the third eye sends novel axonal projections which overlap those of one or both normal eyes. An epigenetic process of competition leads to a self-sorting of the two eyes into non-overlapping regions. At adulthood, such three-eyed frogs show optical dominance columns, like those of binocular mammals (Constantine-Paton and Law 1978). The three-eyed frog develops dominance columns as a side-effect of a general, Hebbian process of vertebrate brain self-wiring.

Let us now ask whether optical dominance columns are an adaptation for binocular vision. Certainly in the frog they are not: such columns only occur in the laboratory after eye transplants, in a species that normally lacks binocular vision, so such columns can hardly have been selected for. In the cat or human, the answer is not so clear. The frog example suggests that the general capacity to form dominance columns was present in our pre-binocular vertebrate ancestors, and simply anatomically rearranging the eye locations would have been sufficient for dominance columns to emerge in the brain. This does not, of course, rule out the possibility that other, more fine-grained features of optical dominance columns are adaptations for binocular vision. But the developmental process that generates dominance columns cannot, itself, be termed an adaptation for something it predated by millions of years. At best, the Hebbian process of self-sorting might be termed a “pre-adaptation” for binocular vision (taking pains to strip this term of any connotation of foresight on the part of natural selection). More perspicaciously, we might adopt Gould & Vrba’s term “exaptation” for the process by which the capacity to develop dominance columns was exploited, evolutionarily, by binocular animals like cats or people. Then the question is whether, after this exaptive event, any further fine-tuning occurred. If so, it would be the fine-tuning, rather than dominance columns themselves, that would correctly be termed an adaptation.

To the relevance of this example to human language, consider the brain circuits involved in reading and writing. Writing is clearly a recent cultural development. Alphabetic writing appears to have been invented only once in the history of our species, a few thousand years ago. Given this short timespan, modern human abilities to read and write can hardly be considered adaptations. Nonetheless, there are several reports of a fascinating condition termed alexia without agraphia (“pure alexia”), in which a brain-damaged patient loses the ability to read but retains the ability to write. Such patients can write individual words, and even take dictation, but afterwards are unable to read what they have written. General visual and manual abilities remain intact. Although rare, this syndrome has been repeatedly reported in the neurological literature (Geschwind and Kaplan 1962; Geschwind 1970), and similar cases have been reported for written music (Brust 1980). Alexia without agraphia provides a cautionary tale for those tempted to assume that specific brain “modules” are adaptations. Finding a discrete brain region or circuit whose destruction impairs reading, but leaves writing intact, is no demonstration that these skills represent genetically determined, functionally specialized adaptations.

The processes of neural development, skill acquisition and learning (in some suitably broad sense) all clearly enable our brains to fine-tune their structure and computational behaviour to the tasks we face. As a result, the fact that some particular mechanism is well-suited to the performance of a particular task, in the adult brain, is not itself evidence of adaptation. These facts greatly complicate any reverse engineering approach to cognitive capabilities, for the details of the mechanism, however “functional”, do not provide unambiguous indications of past adaptive history. Without a detailed understanding of the neural development of such functions, we simply cannot know whether the source of “good design” is evolutionary (adaptation to past selective processes) or ontogenetic (developmental tuning to problems faced by the individual organism). In most cases, both will play a role, and thus the function of any given circuit will represent multiple layers of adaptations and constraints, from many different evolutionary epochs, including adaptations, “ghosts of adaptations past”, and current ontogenetic history. In some cases (such as reading and writing) we expect there to be no truly adaptive component at all. In many others (such as music) it is very difficult to say. In the case of language, we can confidently state that the possession of language is beneficial to any contemporary human, and has been so for millennia, but this does not allow us to say which aspects of language are targeted adaptations (much less what function they served in a Pleistocene EEA), and which are simply exaptations, or correlated traits resulting from the rich history of constraints borne in every mammal or primate brain.

In summary, the previous sections show that, for any given neural circuit or cognitive mechanism, considerable research is required to know whether it constitutes an adaptation for the task(s) it is used to perform. Indeed, profitable inquiry will require that we refrain from discussing whole mechanisms as adaptations. Instead, we need to treat finer aspects of cognitive mechanisms, and particularly *novel* aspects of these mechanisms, as potential adaptations. Just as we can treat the length of the giraffe’s neck, but not its vertebral count, as an adaptation for browsing, we can ask whether certain aspects of syntax processing or speech production are adaptations for language, but not the mechanism as a whole. Syntax processing surely piggybacks upon ancient perceptual mechanisms, just as speech production must build on a prior foundation of motor control circuitry. Those bootstrapped neural/cognitive precursors are not themselves adaptations for syntax or speech (cf. Fitch 2011a).

### Language Evolution: Saltation, Continuity and Pre-Adaptation

**Fig. 3:**
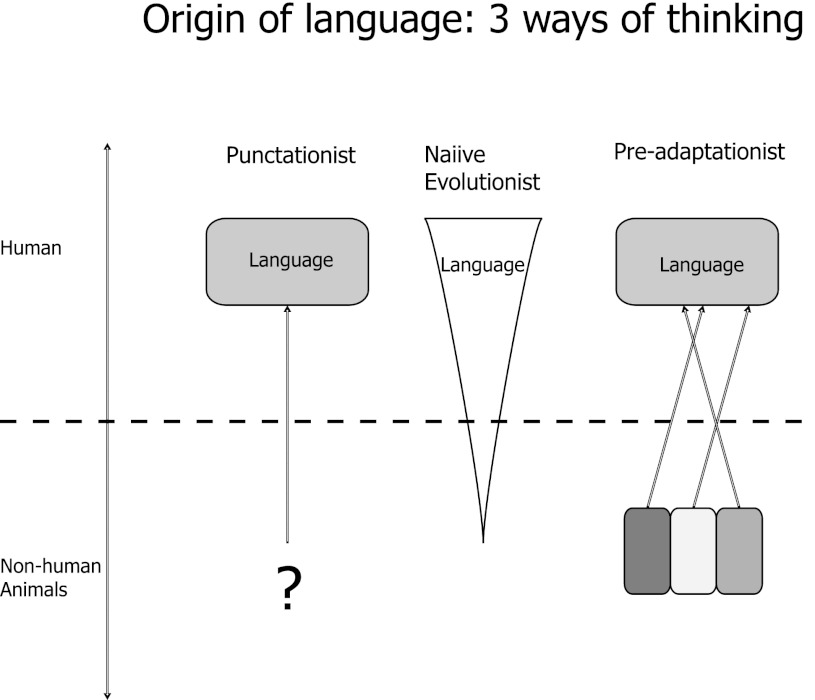
Three Schematized Approaches to Language Evolution The "puctuationist" approach posits a trait appearing de novo, without functionally-relevant precursor traits; the "naïve evolutionist" perspective assumes evolutionary continuity of function, and gradual expansion of the trait; the "pre-adaptationist" perspective posits a novel recombination of pre-existing traits, perhaps with expansion

Clearly, discussions of adaptation in language evolution require us to distinguish cognitive adaptations from constraints on precursor mechanisms. As a step in this direction, Kazuo Okanoya (Okanoya 2007, 2010) has helpfully outlined three common perspectives on language evolution, depicted in Fig. 3, which he dubbed “naïve evolutionist”, “punctuationist”, and “pre-adaptationist” viewpoints. The figure schematically represents changes that led to language during recent human evolution (since our last common ancestor with chimpanzees, roughly six million years ago).

- *Naïve Evolutionist:* The first perspective stresses continuity in a communication system over human evolutionary history. Although the “precursor” system is typically considered to be primate vocal communication (e.g., Hockett and Ascher 1964; MacNeilage 1998; Dunbar 2003), many scholars have argued instead for continuity in gestural communication (Hewes 1973; Armstrong et al. 1995; Corballis 2003; Arbib 2005; Tomasello and Call 2007). But in all cases, the idea is that this precursor system became richer and more elaborate, gradually approaching the complexity of modern human language, with no abrupt changes or truly novel features being posited. Those advocating such models often cite the unyielding gradualism championed by Darwin in support of their viewpoint.
- *Punctuationist:* The second perspective stresses the differences between language and all other known communication systems, and posits a saltationist biological origin for these novel features (e.g., Berwick 1997; Tattersall 1999; Chomsky 2010). From this perspective it is pointless to seek the precursors of language in vocal or gestural communication, since the computations underlying language are of a wholly different sort and are not tied to any particular output modality. Indeed the origin of these features may have been in the context of private thought, rather than having anything special to do with communication (Chomsky 2010). While this punctuationist perspective does not deny the many areas of biological continuity between humans and other animals, it singles out certain aspects of language that seem truly novel, and seeks to explain them discontinuously. It should be noted that there is nothing anti-evolutionary in such a perspective, and there is a long tradition in evolutionary biology of seeking discontinuous origins for novel traits (e.g., Bateson 1894), and some of these ideas are being profitably reinterpreted in modern terms (cf. Gould 2002).
- *Pre-Adaptationist*: This perspective is favored by Okanoya, who assumes a multi-component approach to language evolution, involving recent innovations in signal, syntax and semantics. Increasingly, scholars interested in language evolution appear to be moving away from either extreme pole of continuity or saltation, to acknowledge the likelihood that some aspects of language are best treated as continuous developments or elaborations of ancient traits shared with other species (e.g. vocalization), while others may constitute true novelties that arose since our divergence from chimpanzees (e.g. recursive syntax). This possibility is obviously inherent in a multi-component perspective on language: as soon as one accepts the necessity for multiple different biological mechanisms underlying modern language, the possibility that they have separate, and perhaps quite different, evolutionary histories follows naturally. A core feature of this multi-component perspective is that it *denies* any one feature of language as central, but instead posits that language required a confluence of multiple mechanisms, each on of which may have had quite different precursors in our pre-linguistic ancestors. Okanoya has dubbed the perspective that focuses on this confluence of features, diagrammed in Fig 3C, the “pre-adaptationist” viewpoint.

The key question, from this perspective, is: how did our ancestors move from a species whose vocal communication system consisted of a set of biologically-given calls capable of expressing a small fraction of what the organism knows, to the nearly unbounded system of expression that we humans take as our birthright? One immediate question this raises concerns speech, and particularly vocal learning. Since all modern humans use speech, in the auditory/vocal domain, as the default output signal, we need to ask how humans evolved this capacity for vocal learning, which characterizes humans and not other apes (Nottebohm 1975, 1976; Janik and Slater 1997). But posing this question makes no assumptions that the “core feature” of language is vocal or spoken, or that there is any necessary connection between language and ape vocal communication. Indeed, Okanoya’s pre-adaptationist perspective gives equal weight to mechanisms underlying complex syntax and semantics, stressing that *all* of these factors had to come together in human evolution to yield spoken language. The major advantage of Okanoya’s perspective is its clear recognition that, for human language to be possible, multiple factors had to fall in place.

### Multiple Origins of “Universal Grammar”

Okanoya’s pre-adaptationist perspective invites a profitable re-interpration of the concept of Universal Grammar in terms of biological constraints. There is a long tradition in linguistic research of referring to the constraints on the system children use to acquire language as “Universal Grammar” (Chomsky 1965, 1990; Montague 1970; Bierwisch 2001; Nowak et al. 2001; Jackendoff 2002; Yang 2004; Fitch 2011b), and an almost equally long tradition of vehemently rejecting this term (Deacon, 2003; Tomasello 2005; Van Valin 2008; Chater et al. 2009; Evans and Levinson 2009). A decade ago we (Hauser et al. 2002) suggested that one reason that this debate has raged so long, and with so little resolution, is that different people take the term “language” to designate different things. We further suggested that by identifying the many aspects of language that *do* have precursors or relatives in other species (termed the “faculty of language in a broad sense” or FLB), we can employ the comparative approach to explore these aspects of language. Finally, we suggested that the set of components of language constituting true novelties, unique to both our species and to language, is quite small. We dubbed this set the “faculty of language in a narrow sense” or FLN, and hypothesized that it is perhaps limited to linguistic recursion, or possibly even an empty set. In plain words (cf. Fitch et al. 2005), this means that most of what has been hypothesized to be part of “Universal Grammar” is part of the FLB and derives from older mechanisms (e.g. constraints on general cognition) or processes (e.g. constraints on neural development), rather than being novel to either humans or language.

I would like to make this exaptationist proposal more explicit, since it has frequently been misinterpreted (cf. Fitch 2011b). Hauser, Chomsky and Fitch proposed that most aspects of language have precursors or related mechanisms in other species (and are thus part of FLB). Many such cognitive mechanisms, although important for language, result from more general developmental and epigenetic processes, evolved long before language or humans arose. In other cases, as for optical dominance columns in three-eyed frogs, traits might appear in language that have no obvious equivalents in other species, but nonetheless result from shared developmental mechanisms (the evo-devo component of this proposal). In any case, many constraints will operate on the modern human language system that have deep roots, and we will be unable to understand how language operates, or why it developed the way it did, without a rich understanding of these previous constraints. This perspective on the biological origins of evolutionary constraints on syntax has recently been persuasively embraced by (Chomsky 2010), making explicit reference to the traditional ideas about constraints discussed above. But it is in no sense incompatible with the notion that many important components of language are adaptations, in some sense. Rather, this perspective requires a broadening of the explanatory hypotheses considered, and a concomitant sharpening of the evidential basis for explanatory arguments (cf. Andrews et al. 2002; Botha 2011).

## Conclusion: Novel Components of Language Result from a Cascade of Exaptations

**Fig. 4:**
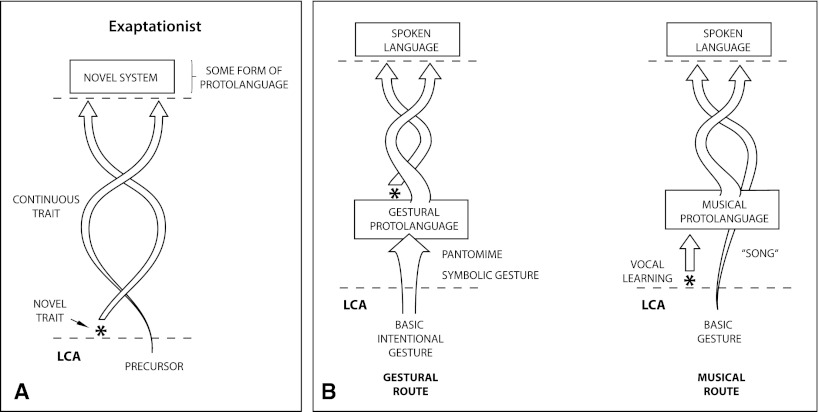
**a** Schematic View of Exaptation in Synergy with Adaptation: the figure illustrates how a novel trait (indicated by the star) can arise, but then interact with preexisting continuing adaptations, to generate a new synergistic trait which is different from either trait. *LCA* Last common ancestor of chimpanzees and humans **b** Two Views of Protolanguage as an Exaptive Cascade: the figures illustrate two hypotheses about a “protolanguage” stage during the evolution of human spoken language. On the *left*, the gestural origins hypothesis posits a gradual expansion of gestural capacities already present in the LCA into a more elaborate gestural protolanguage, with the later addition of vocal learning creating modern spoken language. The musical origins hypothesis posits an early “key innovation” of vocal learning, in the service of song, which provides an early musical protolanguage; this is later combined with continuous cognitive and gestural components to create modern spoken language

I conclude by attempting to draw these various strands together to offer a slight modification and elaboration of Okanoya’s pre-adaptationist perspective, which I dub “exaptationist”. The main difference is that, rather than conceiving of all the component mechanisms of modern language as already being present pre-linguistically in the LCA, albeit in reduced form, I suggest that a cascade of innovations was required, each one creating the pre-conditions for the later ones to be functional and adaptively favored. This conception is illustrated in Fig. 4. The main difference from the pre-adaptationist viewpoint (Fig 3C) is a more explicit focus on the *sequence* in which various pre-adaptations were put to use, and an emphasis on a sequential cascade of exaptations. At each stage of this sequence, a new system of “protolanguage” arose, with its own features and useful in its own right, but lacking others that are present today. In each of these stages, some chunks of pre-existing biology was co-opted or “hijacked” and put to new use. Each exaptive event created a new set of selective pressures on subsequent hominids, leading to the cascade of exaptations that yielded our present full language.

I have argued that at least three different sets of novel abilities, in the domains of vocal *signaling, syntax*, and *semantics*, had to evolve in the six million years since our evolutionary history diverged from that of other apes (Fitch 2005, 2010). While different scholars may emphasize one or the other of these features, the comparative data make clear that all three sets of innovations differentiate us from chimpanzees, bonobos, and our other ape cousins. Any of these three may represent a true novelty, in the sense of a “key innovation” (Liem 1973) or “breakthrough adaptation” (Lovejoy 1981) that lacks precursors and makes a central difference to subsequent evolution. For example, vocal learning certainly represents a key innovation for the production of speech or music. However, it has extensive convergent parallels in other vertebrates, at least some of which share underlying developmental mechanisms (Fitch 2009a). Similarly, human recursive syntax may be quantitatively superior to the basic combinatorial mechanisms observed in sign-trained chimpanzees (Savage-Rumbaugh et al. 1993), but still build upon these as precursors. Alternatively, unbounded Merge may represent a true novelty with no computational precursors (Chomsky 2010), but one that still inherits more general constraints on neural development that structure its functions, and limitations. But in each case some plausible homologs or analogs exist, and it seems premature to decide such points without further investigation.

The exaptation cascade perspective makes no commitments to the particular order in which different mechanisms were exapted and selected. To illustrate this, Fig 4b and 4c contrast the “musical protolanguage” hypothesis of Darwin with the “gestural protolanguage” hypothesis of Condillac, Hewes and many others (Darwin 1871; Condillac 1747/1971; Hewes 1973). The first model stresses a discontinuity, between vocal learning and the lack thereof, as an early event in language evolution. The second stresses a continuity, between gestural communication in apes and humans, as the scaffolding for innovations in symbolic and syntactic systems, with vocal learning as a later and relatively inconsequential innovation. The differences between these two models are explored in detail elsewhere (Fitch 2010) and will not be discussed here. The point is simply that one can adopt the exaptationist perspective without making any premature commitments about what exactly was exapted, or in what order.

From this exaptationist perspective, the crucial questions in language evolution concern:

1. *Novelties*: What cognitive abilities needed to evolve since our divergence from chimpanzees?
2. *Mechanisms*: What genetic, developmental and neural/computational mechanisms underlie these abilities?
3. *Precursors*: How are these mechanisms related to pre-existing mechanisms, both in function and development? What constraints do they inherit from those precursors?
4. *Interactions/Synergies*: How do these novel mechanisms interact with each other, and how do they exploit and interact with other, unchanged mechanisms?
5. *Computation*: What computational and algorithmic function(s) does each mechanism serve, in the present day, and to what extent are these computational functions shared, either with precursor mechanisms, or more broadly with other species and other aspects of cognition?

The question “is mechanism *x* an adaptation?” is absent from this list, because the over-simplistic sense of “adaptation” often deployed in discussions of cognitive evolution (where traits either are, or are not, adaptations) is of very limited use in discussing language evolution. My omission is not due to any hostiliy to the concept of adaptation by natural selection: like any biologist I see adaptation as a key component of evolutionary explanation. Rather, my skepticism results from a *respect* for adaptation as a concept, and a recognition of acute limitations on our scientific ability to validate that concept in the case of human cognitive abilities. If it is difficult to tease out and understand the target of selection in the case of morphological adaptations like bipedalism or our vocal tract, where the physics and physiology are well-understood, how much more challenging will that same question be for cognitive traits, where neither the basic computational issues nor the adaptive problem space are well understood. Although we can certainly hope for progress on the five issues above, questions about adaptation may remain forever intractable. If this is correct, the cognitive sciences as a whole will be well-served by a continued focus on mechanisms, precursors, and computational function (which, after all, represents the current norm). Interest in cognitive evolution should lead to a broadening of perspective, more fully embracing comparative cognition. This will enable scientists to better delineate and understand the many constraints on cognition and learning that we have inherited.

Focusing on empirically inaccessible questions of adaptation, as advocated by some evolutionary psychologists, simply diverts attention from these central empirical objectives. Questions about adaptations should of course continue to be asked: they may provide an enlightening (and entertaining) source of intuitions and new hypotheses. But, for reasons that I hope are now clear, such questions are unlikely to be answered definitively. In stark contrast, I think that the questions listed above can, and should, be answered via increased empirical research. In doing so we will reach a much deeper and more satisfactory understanding of language and its evolutionary origins, one which incorporates the many complex constraints on development, and avails itself of the richer and more pluralistic perspective on evolution that has been so beautifully unveiled by the last two decades of developmental research.
